# The overturn of *Roe v Wade*: Google searches for teratogenic medications following the U.S. Supreme Court ruling

**DOI:** 10.1097/JW9.0000000000000139

**Published:** 2024-04-02

**Authors:** Ambika Nohria, Deesha Desai, Elizabeth J. Klein, Maryanne M. Senna, Crystal Aguh, Ronda S. Farah, Lindsey Bordone, Loren D. Krueger, Natasha Mesinkovska, Donna Cummins, Kristen I. Lo Sicco

**Affiliations:** a The Ronald O. Perelman Department of Dermatology, New York University Grossman School of Medicine, New York City, New York; b University of Pittsburgh School of Medicine, Pittsburgh, Pennsylvania; c Department of Dermatology, Lahey Hospital & Medical Center, Burlington, Massachusetts; d Department of Dermatology, Johns Hopkins School of Medicine, Baltimore, Maryland; e Department of Dermatology, University of Minnesota, Minneapolis, Minnesota; f Department of Dermatology, Columbia University Irving Medical Center, New York City, New York; g Department of Dermatology, Emory University School of Medicine, Atlanta, Georgia; h Department of Dermatology, University of California Irvine, Irvine, California; i The Dermatology Centre, Salford Royal Hospital, Northern Care Alliance NHS Foundation Trust, Manchester, United Kingdom

**Keywords:** abortion, Google trends, isotretinoin, teratogenic

**What is known about this subject in regard to women and their families?**

- The *Dobbs v Jackson Women’s Health* Supreme Court decision, which ended nearly 50 years of federal abortion protection, had a profound impact on women’s health care decisions in the United States.
- This decision prompted many women to reconsider their reproductive care choices, for example, regarding medical contraceptive methods.
- The decision also prompted many health care providers and policymakers to reevaluate the intersection of legal decisions and women’s health care choices.

**What is new from this article as messages for women and their families?**

- The article highlights the significant impact of the U.S. Supreme Court’s ruling in *Dobbs v Jackson Women’s Health* on patients’ health care considerations beyond reproductive care.
- By analyzing Internet search patterns for teratogenic dermatologic medications, we demonstrate evolving attitudes and increased searches in the United States compared with England, indicating potential influences of federal laws on patients’ medical concerns.
- Women should be encouraged to discuss medical decision-making with their dermatologist in the context of the reproductive legal landscape that they live in.

## Dear Editor,

On June 24, 2022, the U.S. Supreme Court (SC) made a landmark ruling in *Dobbs v Jackson Women’s Health*, ending nearly 50 years of federal abortion protection by declaring that the U.S. Constitution does not confer rights to abortion.^1^ Dermatologists and patients alike have raised concerns regarding the potential impact of abortion bans on access to teratogenic therapies used for dermatologic conditions, such as isotretinoin and methotrexate.^2^ For example, a young woman prescribed isotretinoin for acne may hesitate due to new limitations on abortion access. We aimed to evaluate these concerns among the U.S. public and compare them with those in England, where abortion is legalized for up to 24 weeks nationwide under the Abortion Act of 1967.^3^ To assess this, we analyzed Internet search patterns regarding teratogenic medications around the time of the U.S. SC decision.

Using Google search trends, we quantified searches for isotretinoin (Accutane in the United States, Roaccutane in England) and teratogenic. Google trends provide a relative search volume (RSV) score ranging from 0 to 100, indicating the popularity of a term compared with all searches within a specific timeframe and location. Our analysis spanned 2 periods: May 20, 2022–June 17, 2022 and June 26, 2022–July 24, 2022, excluding the week of the SC decision. Additionally, we examined search data from June 23, 2021–June 23, 2022 and June 25, 2022–June 25, 2023, to understand trends surrounding the SC decision. Statistical significance was determined using paired *t* tests, with *P* < .05.

In the United States, searches for Accutane significantly increased from the month before to the month after the SC decision (*P* = .0026), whereas no significant increases were observed in England. Comparing RSV between the United States and England, searches were significantly higher for isotretinoin in the month before the SC decision (*P* = .002) and for isotretinoin and teratogenic in the month after (*P* = .00 and *P* = .005) (Table 1).

**Table 1:** Relative search volume (RSV) before and after Supreme Court ruling

Search term, country of search	Average RSV May 20, 2022–June 17, 2022	Average RSV June 26, 2022–July 24, 2022	Average RSV June 23, 2021–June 23, 2022	Average RSV June 25, 2022–June 25, 2023
Isotretinoin				
USA	57.28	60.45	83.57	76.77
England	35.03	29.48	64.69	71.40
Accutane				
USA	72.57	62.72	82.58	72.75
Roaccutane				
England	45.79	55.51	16.81	56.80
Teratogenic				
USA	32.48	38.69	56.85	61.53
England	20.76	13.34	38.98	35.21

From the year before to the year after the SC decision, significant increases in searches for isotretinoin, Accutane, and teratogenic were observed in the United States (*P* = .00, *P* = .00, and *P* = .04), while England saw significant increases in searches for isotretinoin and Roaccutane (*P* = .0058 and *P* = .00). RSV for isotretinoin in the United States was significantly higher than in England both before and after the SC decision (*P* = .00 and *P* = .00), as were searches for teratogenic (*P* = .00 and *P* = .00) (Table 1).

Changes in RSV surrounding *Dobbs v Jackson* may indicate evolving attitudes toward these medications and increased hesitation regarding teratogenic dermatologic medications. Notably, higher U.S. search volumes for isotretinoin and teratogenic compared with that in England suggest that federal laws may influence patients’ medical concerns. Limitations of this study include confounding factors influencing RSV, such as fluctuations in prescription rates during specific periods.

Dermatologists must consider the impact of regional reproductive laws on patients’ treatment decisions. Similar trends to those observed during the SC decision may reappear as courts continue to address reproductive rights. Dermatologists can use this data to conduct further research on patient perception of therapeutics in a post *Dobbs v Jackson* landscape.

## Conflicts of interest

The authors made the following disclosures: K.L.S. has been an investigator for Regen Lab and is an investigator and consultant for Pfizer. K.L.S. is a consultant for Aquis and is on the board of directors for the Scarring Alopecia Foundation. L.D.K. is a consultant for Janssen and is on the board of directors at Skin of Color Society. A.N., D.D., M.M.S., R.S.F., L.B., C.A., E.J.K., N.M., and D.C. have no conflict of interest to declare.

## Funding

None.

## Study approval

N/A

## Author contributions

AN, DD, and EJK: Contributed to the design and development of this manuscript. MMS, CA, RSF, LB, LDK, NM, DC, and KLS: Contributed to the design and editing of this manuscript.
